# Coexistence of ipsilateral acute‐on‐chronic subdural hematoma and acute extradural hematoma: A case report

**DOI:** 10.1002/ccr3.7684

**Published:** 2023-07-10

**Authors:** Jawad Basit, Saad Javed, Faizan Shahzad, Eesha Yaqoob, Sajeel Saeed, Ayush Anand

**Affiliations:** ^1^ Department of Medicine Rawalpindi Medical University Rawalpindi Pakistan; ^2^ Department of Neurosurgery Rawalpindi Medical University Rawalpindi Pakistan; ^3^ Department of Sociology Pir Mehr Ali Shah Arid Agriculture University Rawalpindi Pakistan; ^4^ B. P. Koirala Institute of Health Sciences Dharan Nepal

**Keywords:** case report, extradural hematoma, neurosurgery, subdural hematoma

## Abstract

Chronic subdural hematomas are typically observed in elderly patients receiving antithrombotic and/or anticoagulant therapy. In contrast, acute subdural and extradural hematomas are often observed in young people with traumatic brain injury. The coexistence of ipsilateral chronic subdural and extradural hematomas is rare. Depending on the Glasgow Coma Scale and neuroimaging findings, early surgical intervention is mandatory, as seen in our patient. Early surgical evacuation of a traumatic extradural and chronic subdural hematoma should be done. Also, antithrombotic drug use can lead to chronic subdural hematoma.

## 1 INTRODUCTION

Extradural hematomas (EDH) and subdural hematomas (SDH) are extra‐axial hemorrhages. Acute EDH is often observed in young people after traumatic brain injury.^1^ In contrast, chronic SDH is more commonly observed in elderly patients following minor or unperceived traumas.^1^ Chronic SDH can be attributed to various factors, such as anticoagulants, antiplatelet drugs, chronic alcoholism, and intracranial hypotension.^2^ Acute‐on‐chronic SDH may occur; however, the coexistence of ipsilateral EDH is rare.^3^ Herein, we present the case of ipsilateral traumatic EDH and ipsilateral acute‐on‐chronic SDH in an elderly male.

## 2 CASE REPORT

A 65‐year‐old male presented to the emergency with loss of consciousness following a road traffic accident. The patient was in the driving seat, not wearing a safety belt. The collision details and the exact timing of the injury could not be established. Personal history revealed arterial hypertension, for which the patient was taking amlodipine and valsartan, and ischemic heart disease, for which the patient was taking aspirin and clopidogrel. His personal history, family history, and psychosocial history were unremarkable.

The Glasgow Coma Scale (GCS) score was 7 at the presentation, and a dilated left‐sided pupil was observed. His vitals were a pulse rate of 58 per minute, blood pressure of 140/90 mm of Hg, and respiratory rate of 35 cycles per minute. A detailed neurological examination was not possible in the emergency setting.

**FIGURE 1:**
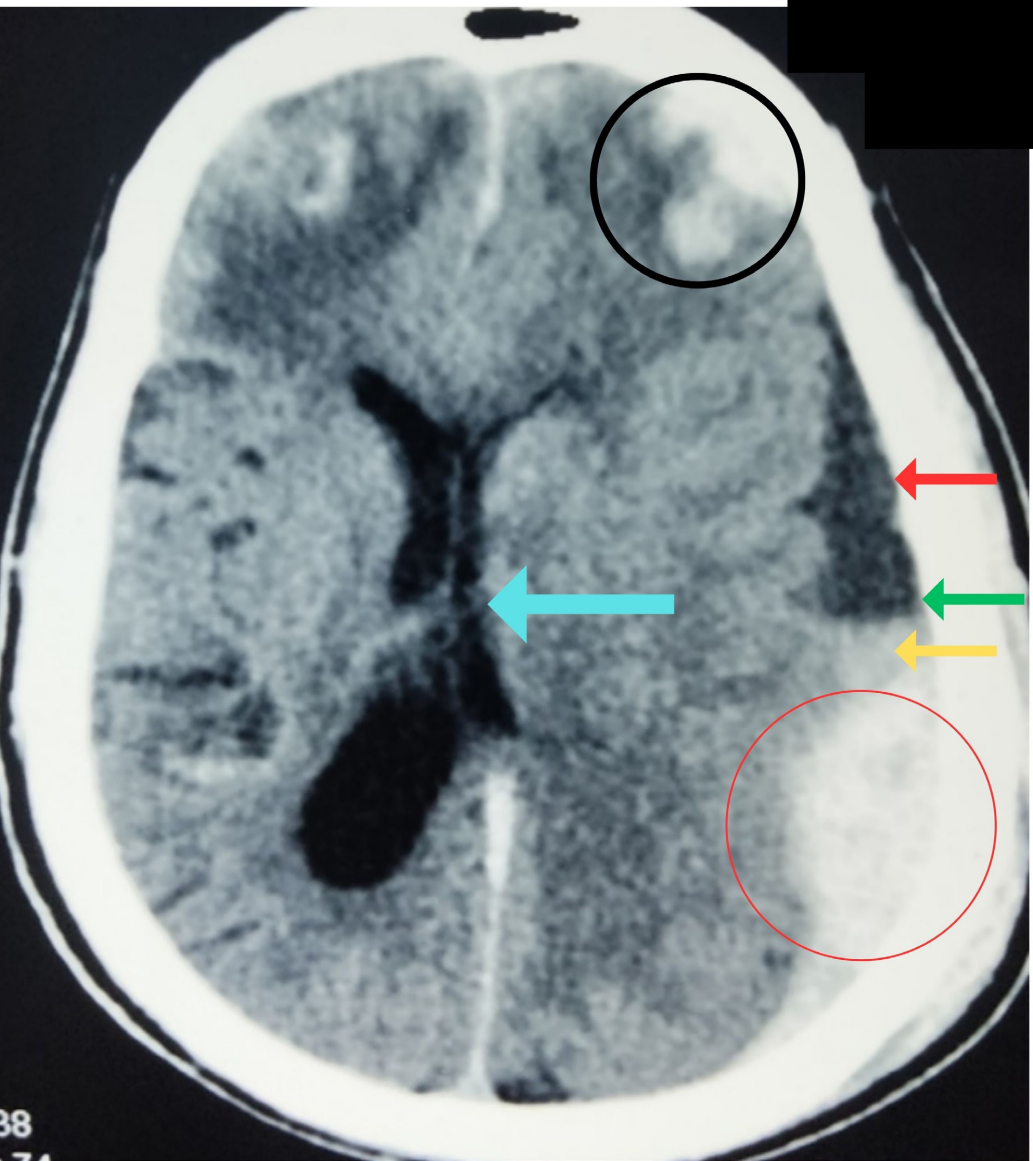
A computed tomography scan of the head showing contusions (black circle), a hypodense area (red arrow) with a fluid–fluid level (green arrow) and a hyperdense area below (yellow arrow) it with rightward midline shift (blue arrow) suggesting acute‐on‐chronic subdural hematoma, and a hyperdense area suggesting extradural hematoma (red circle).

**FIGURE 2:**
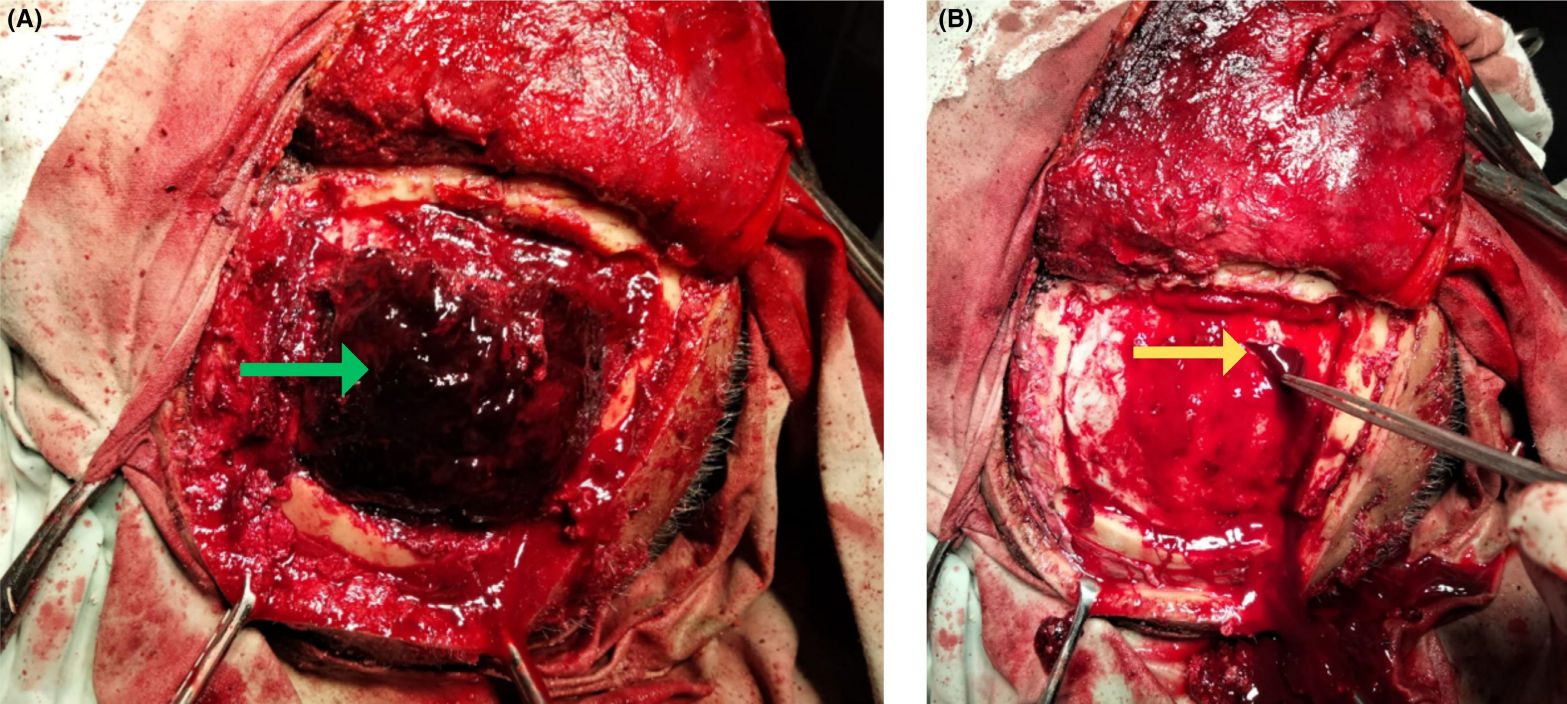
(A) Extradural hematoma (green arrow). (B) Dural incision (yellow arrow) given to drain chronic subdural hematoma with machine oil appearance.

Immediate orotracheal intubation was done, two wide‐bore intravenous lines were opened, and resuscitation was done with 0.9% normal saline. His blood investigations did not show any abnormalities. Coagulation profile tests were not done as the patient needed emergency surgery. Following this, we did an urgent computed tomography (CT) scan of the head, which revealed subdural hematoma, extradural hematoma, frontal contusions, and a rightward midline shift with no bone fractures (Figure 1). The volume of EDH was approximately 40 mL, and acute‐on‐chronic SHD was around 35 mL. The midline shift was about 5 mm. After a neurosurgery consultation, we did an urgent emergency surgical evacuation of both lesions with the same incision and craniotomy. A session with the prime attendants was done to brief the operative details and the risk of general anesthesia and surgery as the patient had been taking anticoagulation therapy. After taking high‐risk consent, the patient was moved for surgery under cover of packed red blood cells and platelets to avoid any unwanted surgical events. The scalp was opened in layers, and a reverse question mark incision was made. A 6‐burr hole craniotomy was made. There was a massive extradural hematoma, approximately 50–60 mL in volume (Figure 2A), in the left temporoparietal region and a subdural hematoma (Figure 2B) in the left frontotemporoparietal region. The EDH was evacuated in a single attempt. The bleeding was from the middle meningeal artery. The bleed and hemostasis were secured. After the EDH evacuation, an SDH evacuation was planned. One dural incision was given 2 cm away from the anterior margin of craniotomy, and one was given 2 cm away from the posterior margin. Upon giving the incisions, SDH poured out. It had a machine oil appearance. It was thoroughly washed with 3 L of warm saline via an 8 Fr Nasogastric tube. A drain was placed, via the slit, in the subdural space, and the dura mater was stitched loosely to avoid any subdural recollection. Hemostasis was rechecked. Bone was placed back, and the scalp closed in reverse order.

Postoperatively, the patient was shifted to the Intensive care unit, extubated after 1 day of stay, and then shifted to the surgery ward. A repeat CT scan of the head was done after 2 days, which revealed adequately evacuated SHD and EDH with minimal residual collection. Hence, the drain was removed on the second postoperative day. The patient did well on monthly follow‐ups, and no adverse events were reported.

## 3 DISCUSSION

EDH is reported chiefly in head trauma cases caused by road traffic accidents in men.^1^ EDH is mainly seen in young adults.^1^ However, it can be reported less commonly in an elderly male, such as in our case. Usually, the middle meningeal artery is involved, although venous bleeding may also be a source.^1^ In our patient, the middle meningeal artery injury led to EDH. In contrast to EDH, chronic SDH is reported mainly in elderly males.^1^ SDH risk factors include using anticoagulants, antiplatelet drugs, chronic alcoholism, and intracranial hypotension.^2^, ^4^ Hence, a chronic subdural hematoma can be attributed to our patient's long‐term use of antithrombotic drugs.

**FIGURE 3:**
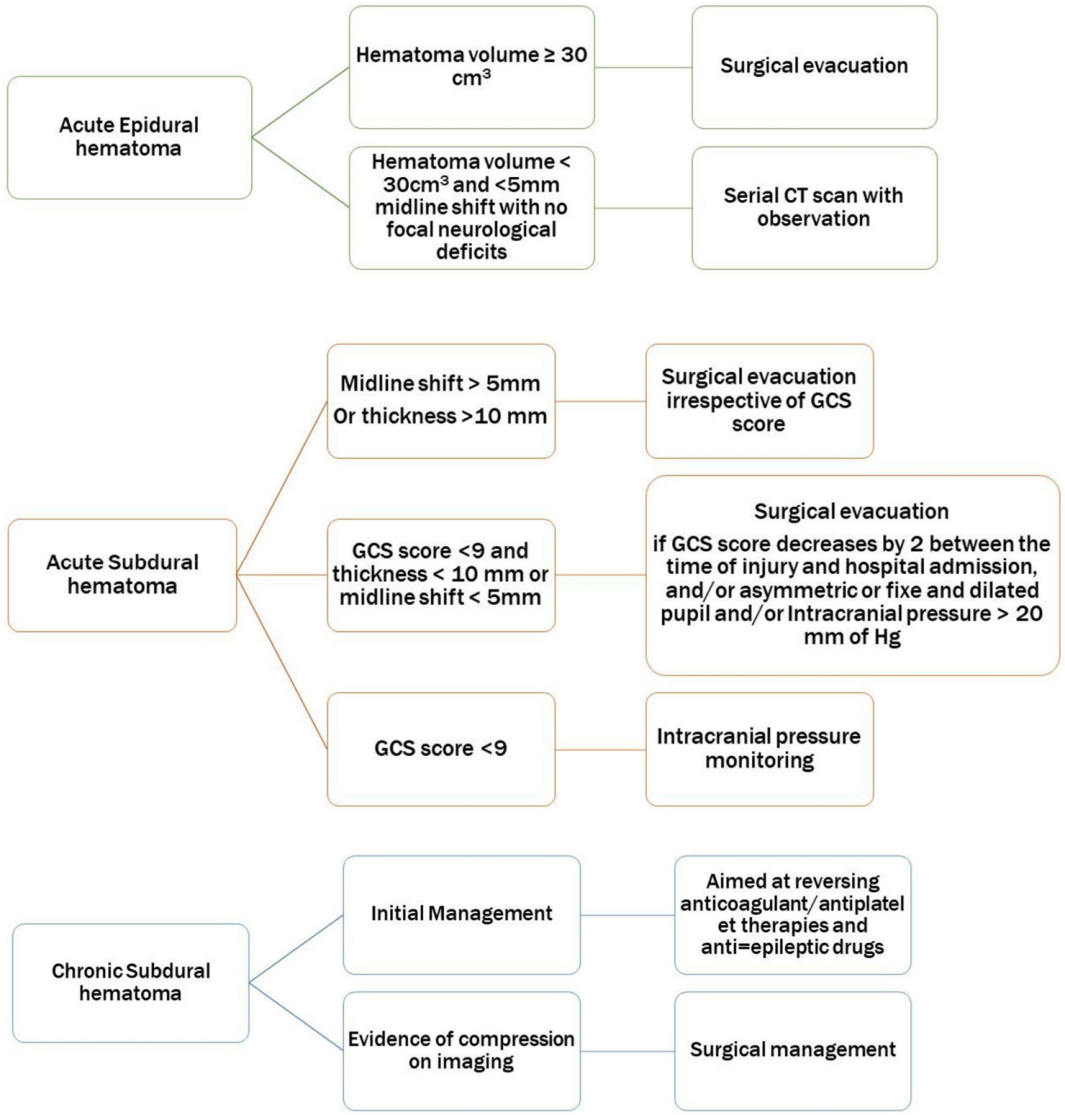
Management algorithm for acute epidural and subdural hematoma and chronic subdural hematoma.

In head trauma cases, a head CT scan is the most widely used imaging modality. Acute EDH on CT appears as a crescentic or biconvex hyperdense collection in extradural space.^5^ Extent of EDH is confined due to sutures between the skull and the dura mater.^5^ Acute SDH presents as a hyperdense collection in the subdural space, and bony sutures do not limit its extent.^5^ Depending on the internal architecture, the acute‐on‐chronic SDH can present as homogenous, laminar, separated, and trabecular type.^6^ The separated type can be identified as a high‐density collection below the lower‐density one with a clear margin between them and the high‐density collection showing recent bleeding.^6^ We did an urgent head CT scan, which revealed acute EDH, acute‐on‐chronic SDH (separated type), and frontal contusions with rightward midline shift. In addition to diagnosis, a non‐contrast CT scan of the head can quantify the hematoma volume, which can guide the intervention approach and reflect the prognosis along with the GCS score (Figure 3).^7^

Treatment options for acute traumatic EDH can vary based on the GCS score and hematoma volume.^8^ EDH with a volume of less than 30 cm^3^ and less than 5 mm midline shift in patients with GCS more than eight without focal neurological deficits can be managed non‐operatively with serial CT scans and vigilant observation.^9^ Early surgical evacuation is mandated in patients with an EDH volume of 30 cm^3^ and more, irrespective of GCS score, leading to an excellent prognosis.^8^, ^9^ Our case was unique as the patient had acute EDH with Acute‐on‐chronic SDH. Usually, acute SDH with a thickness greater than 10 mm or midline shift of more than 5 mm on head CT should be managed with surgical evacuation, irrespective of GCS score.^10^ Patients with chronic SDH can be managed with surgical and medical approaches. However, the evidence behind these approaches' applicability to clinical decision‐making is sparse. Initial management includes reversing anticoagulant/antiplatelet therapies and anti‐epileptic drugs.^7^ Surgical intervention is advised in symptomatic cases with evidence of compression on imaging in SDH.^7^, ^11^ Also, placing a postoperative drain can decrease the risk of recurrence of chronic SDH.^11^ Hence, as required in our case, we did a surgical evacuation of chronic SDH after EDH evacuation and placed a postoperative drain to reduce the recurrence of SDH. The early surgical intervention in our patient led to rapid symptomatic improvement, and no symptoms were reported on monthly follow‐ups.

## 4 CONCLUSION

Our case is unique as the patient presented with acute traumatic EDH and coexisting acute‐on‐chronic SDH secondary to antithrombotic use. Surgical intervention is planned in these patients considering the GCS score, symptom status, and hematoma volume. If the acute EDH volume exceeds 30cm^3^, early surgical evacuation is required regardless of the GCS score. Acute‐on‐chronic SDH with evidence of compression on imaging should be managed with early surgical evacuation. Furthermore, a postoperative drain can decrease the risk of recurrence.

## AUTHOR CONTRIBUTIONS

**Jawad Basit:** Conceptualization; data curation; investigation; project administration; supervision; writing – original draft; writing – review and editing. **Saad Javed:** Conceptualization; data curation; investigation; project administration; supervision; writing – original draft; writing – review and editing. **Faizan Shahzad:** Conceptualization; writing – original draft; writing – review and editing. **Eesha Yaqoob:** Conceptualization; writing – original draft; writing – review and editing. **Sajeel Saeed:** Conceptualization; writing – original draft; writing – review and editing. **Ayush Anand:** Conceptualization; data curation; investigation; project administration; supervision; writing – original draft; writing – review and editing.

## CONFLICT OF INTEREST STATEMENT

The author(s) have no conflict of interest to declare.

## CONSENT

A written informed consent was taken from the patient based on the journal's policy.

## Data Availability

All data pertaining to this case is made available within the manuscript.
